# Topics of Public Interest

**Published:** 1905-04

**Authors:** 


### TOPICS OF PUBLIC INTEREST.

### Canal Zone Sanitation.

### The Panama Problem.

**Report to the Government by Dr. Charles A. L. Reed, on the Status of Sanitation in the Canal Zone and in the Cities of Panama and Colon.**

WASHINGTON, D. C., March 2, 1905.

*Honorable William H. Taft, Secretary of War.*

DEAR SIR:—Pursuant to your request I have the honor herewith to submit the report of my observations relative to the status of sanitation and of the sanitary department in the canal zone and in the cities of Colon and Panama.

I arrived at Colon on the 7th of February and sailed from that city on the 22d of the same month, thus affording me fifteen days in which to study, with more or less care, the conditions of organisation and the details of administration as they relate to the public health interest.

SANITARY STAFF EFFICIENT.

I was given every facility in this regard by General Davis, the Governor of the Zone; by Mr. Wallace, the Chief Engineer; by Colonel Gorgas, the Chief Sanitary Officer; as well as by his associates, Major La Garde, Lieutenant Lyster, and Dr. Carter. As a result of this investigation I became impressed with the efficiency and the zeal of the sanitary staff; with the fact that very much has been accomplished in the way of sanitation under exceedingly adverse circumstances; that much remains to be done which cannot be done unless better facilities are afforded; and that very much more ought to be done and would have been done if the facilities had been properly furnished.

I was forced to this conclusion, not only by what I saw and heard while on the Isthmus, but by a careful study of the published proceedings of the Isthmian Canal Commission, by a study of the laws of the canal zone formulated by that commission, and by a careful consideration of their first annual report, submitted under date of December 1, 1904.

. . . . . . .

THE ORGANISATION OF THE HEALTH DEPARTMENT.

At the meeting of the commission, held at Ancon, August 28, 1904, Mr. Grunsky, as the committee on a proposed health department, presented a report which began by stating that "after repeated conferences with" Colonel Gorgas and with practically the entire sanitary staff, "it has been agreed," but which should have stated that "in certain important particulars Mr. Grunsky has agreed with himself"; for, as a matter of fact, much of the report was formulated over the respectful protest of the medical men who were invited to the conference. By this report the commission, more especially Mr. Grunsky, provided for the creation of a board of health with power to formulate regulations which would become effective only after approval by the commission, or, in cases of emergency, only on the approval of the governor of the canal zone. Thus the chief sanitary officer who had been sent to the zone to clear it up and to make it ready for the actual work of the engineers had his discretion limited to the enforcement of regulations that had first been adopted by the commission or by a board of health; in which latter event it had to be sent generally to Washington to be endorsed by the commission, or, in cases of emergency, it could be approved or rejected by the governor of the zone.

THE EXTRAORDINARY SUBORDINATION OF THE SANITATION STAFF.

It thus came about that the chief sanitary officer, whom and whose department the medical profession had asked to be made largely autonomous, whom and which the President himself had obviously intended should be largely autonomous, was, by the action of the commission, more especially Mr. Grunsky, subordinated to the governor of the zone; to the chief disbursing officer; to the chief of the bureau of material and supplies; to Mr. Grunsky; to the commission; to the Secretary of War; to the President; subordinated in fact in the seventh degree from the original source of authority. And this is the state of affairs on the Isthmus today. One cannot but be impressed with the anomalous condition by which a man of Colonel Gorgas's distinction, the foremost authority in the world in solving the peculiar problems that are connected with sanitation on the Isthmus, is being made subordinate of a whole series of other subordinates who are confessedly ignorant of the very questions with which he is most familiar.

HOW THE MACHINE DOES NOT WORK.

It is interesting to inquire into the working of this wonderful mechanism. Thus, if Major La Garde, superintendent of Ancon Hospital, makes a requisition for supplies, he must make it in due form, take it for approval to the chief sanitary officer, then to the governor of the zone, then to the chief disbursing officer; whence it goes to the commission at Washington; then to Mr. Grunsky as committeeman; then back to the commission; then, if allowed, bids are advertised for; awards are made; the requisition is filled under the supervision of a purchasing agent notoriously ignorant of the character and quality of medical and surgical supplies; the material is shipped to the Isthmus, consigned to the chief of the bureau of materials and supplies, who notifies the disbursing officer, who notifies Colonel Gorgas, who in turn notifies Major La Garde, who applies to the quartermaster,—the boss of a corral,—for transportation, and so much of the stuff as in the judgment of, first, the governor, next the chief disbursing officer, next the commission, next, and more particularly, Mr. Grunsky, ought to be allowed to the superintendent of Ancon Hospital finally arrives or does not arrive at its destination. This is no fanciful picture; it is exemplified in practically every ordinary requisition that goes forward. And what is true of Ancon Hospital is true at Colon, at Culebra, at Miraflores and at all points along the line that require supplies of this description. It is true that in the presence of emergency it is not necessary to send clear to Washington, and certain purchases are permitted and authorised in the open market at Panama, but always, of course, at greatly increased prices.

An instance in point occurred a few days before my departure from Ancon: A woman in the insane department was delivered of a child; her condition was such that she could not nurse her offspring; the nurse applied to Major La Garde for a rubber nipple and a nursing bottle; he had none,—the requisition of last September had not yet been filled; he made out a requisition, took it to Colonel Gorgas for indorsement, then to Mr. Tobey, chief of the bureau of materials and supplies for another indorsement, then to a clerk to have it copied and engrossed; then a messenger was permitted to go to a drug store and buy a nursing bottle and a nipple, which finally reached the infant two days after the necessity for their use had arisen. The articles ought to have cost not more than 30 cents, but counting the money value of the time of the nurse, of Major La Garde, of his clerical help, of Colonel Gorgas, of Mr. Tobey, of Mr. Tobey's clerks, of the messenger, the cost to the government of the United States was in the neighborhood of $6.75,—all due to the penny-wise and pound-foolish policy of the commission, more especially of Mr. Grunsky.

. . . . . . .

THE SANITARY DEPARTMENT DENIED THE RIGHT TO DECIDE ON THE SUITABILITY OF ITS OWN SUPPLIES.

The commission at Ancon last August adopted a resolution creating a department of the zone government to be known as the "material and supplies department," the chief of which is "charged with the receipt, inspection on the Isthmus, custody, care, shipment, transfer, issue and disposition of all supplies, material, equipage and floating equipment unissued and not in actual use." It is, however, specifically provided that:

> The governor of the Canal Zone, acting for the executive branch of the government of the zone, and the chief engineer, acting for the Department of Engineering and Construction, shall have authority to decide on the suitability of any and all supplies furnished, and their requisitions for such materials as are on the Isthmus shall be promptly filled without reference to a higher authority.

There is nothing said about the sanitary department, and as that which is not included is excluded, it follows that the right very justly accorded to the Engineering Department is denied to the sanitary department as such. It is obvious, of course, that the sanitary department, being made subordinate to "the executive branch of the government of the zone," can get supplies, but subject only to the judgment of the governor, who, by this resolution, is empowered to pass on the suitability of any or all supplies for the Medical Department or the Department of Public Health."

An example of the working of this rule was shown in the recent purchase of an *x*-ray outfit for the Ancon Hospital. The requisition went in several months ago and was pending before the commission, more especially Mr. Grunsky, when the expert who had been specially employed to do *x*-ray work in Ancon, happened in Washington and requested the privilege of selecting the Crookes tubes; the request was peremptorily refused; the expert urged that his knowledge would enable him to make better selections than would be probable of an unskilled purchasing agent, and went so far as to urge as an additional reason the distance to the Isthmus, the time involved in transportation, to say nothing of the expense involved, only, however, to be informed that if the tubes did not suit they could be returned. They came after a long delay, were found worthless and were returned; other ones have not yet been sent to replace them and, as a consequence, Ancon Hospital is today without *x*-ray service, while the salary of the expert goes on. In another instance objectives for a number of microscopes were carefully specified by the chief of the laboratory, who would naturally be presumed to know most about them, but the commission, more especially Mr. Grunsky, assumed to possess superior knowledge of the subject and substituted others, with the result that when they arrived on the Isthmus they, too, were found to be worthless.

The foregoing are but two examples of what is constantly occurring. The matter becomes really very serious when it involves questions of medicines, and in this particular furnishes a strange inconsistency with the laws laid down by the commission for the government of everybody else but themselves. Thus, by the laws of the canal zone, a severe penalty is imposed on druggists and purveyors in general for substituting one medicine for another that may have been ordered, yet instance after instance is coming to the front in which the commission, either through Mr. Grunsky or through an unqualified purchasing agent, is foisting on the medical service remedies other than those specified in the requisitions. This, I believe, holds true in practically every requisition for medicine that has reached Ancon Hospital, and the same principle may probably account for the fact that the majority of all of the requisitions for medical supplies have been either ignored or suppressed by the commission at Washington.

. . . . . . .

CHEAP PHYSICIANS FOR THE ISTHMUS.

The commission in every effort that it has made to secure service of any character on the Isthmus has tacitly acknowledged the unhealthfulness of the region by holding out as an inducement the fact that employes will be furnished free medical treatment, including the service of the hospitals. The fact that medical men in the zone would have much executive work to do, that they would have to deal with large bodies of workmen, and that their duties would require the exercise of trained judgment in a very broad sense, prompted Colonel Gorgas to advise that only relatively mature men be brought to the Isthmus in the capacity of physicians. He advised, furthermore, that the minimum salary to be paid to medical men in the zone be the same as the minimum salary paid in the Army for contract surgeons—namely, $1,800. This plan did not, however, commend itself to the commission, more especially to Mr. Grunsky, who, in the interest of alleged economy, conceived the brilliant scheme of establishing interneships in the hospital of the zone, the incumbents to receive $50 per month, the same salary that is paid to nurses. The verbal justification of the plan offered by Mr. Grunsky, and subsequently adopted by the commission, is that young men will thereby receive a preliminary training in tropical diseases, which is to be accepted by them as part pay for their services, after which, that is, after a year, if they so desire, they will be at liberty to return to the states. But Mr. Grunsky takes pains not to say that the incidental service to be rendered by these internes is to represent the bone and sinew of the medical service on the Isthmus, and likewise fails to make clear how he expects to establish a stable medical service if, after the expiration of a year, his internes are at liberty to return to the North, which they would doubtless do in the absence of inducements to remain on the Isthmus. And what if they should desire to return before the end of a year?

This question brings us face to face with Mr. Grunsky's trap to get cheap medical service for the zone. Once on the Isthmus, these young men, finding themselves on the salaried basis of nurses with incidental expenses that cannot be evaded and that will eat up the last penny of their beggarly stipend, desiring to leave their humilating positions, will find the door closed against egress. It is even today easy for an employe to get to the Isthmus, but it is already exceedingly difficult for him to get away from it. And what is true today will be more emphatically true in the future, a fact that the commission, more especially Mr. Grunsky, takes great care to leave in the background.

But the commission, more especially Mr. Grunsky, holds up the prospect of promotion. This in any event, under Mr. Grunsky's rules, cannot be granted under one year, and then, if granted, which it may not be, the salary is placed at $125 per month, or $300 per year less than the minimum salary paid to contract surgeons in the Army. What is there about the medical service in the canal zone that should render it less entitled to compensation than the same medical service when rendered in the Army? What is there about the personnel of the zone government, about the employes, about the laborers, that they should be furnished with a cheaper grade of medical service than the officers, soldiers and enlisted men of the Army? Why should not the commission, more especially Mr. Grunsky, follow the example of both the Army and the Navy and accept men who have already served interneships in the hospitals of the states, give them decent salaries, and then, after sending them to the Isthmus, give them additional opportunities for the preliminary study of tropical diseases at the hospitals at Ancon and Colon? And why should this not be done at once, that a competent medical staff may be at the Isthmus when the large bodies of workmen shall have arrived? This could not but be gratifying to the chief sanitary officer, whose wishes, however, it would seem need only to be expressed to have them vetoed by the commission, more especially by Mr. Grunsky, in what seems to be a fatuous desire, regardless of consequences to give every detail the impress of his overpowering personality.

### THE UNITED STATES IN CHEAP COMPETITION WITH THE MEDICAL PROFESSION OF PANAMA.

The course of the commission in its endeavor to establish a cheap medical service, naturally enough made up of cheap doctors, which they hope in the future to send to the zone, is not limited in its influence and in its pernicious results to the zone itself. To make this clear, however, it is important to remember that under the truly remarkable plan of organisation of the sanitary department devised by Mr. Grunsky, adopted by the commission and duly engrossed in the laws of the canal zone, it is provided that all persons not employes of the commission, but who may be "sick in the canal zone and in Colon and Panama, should be admitted to the hospitals, but such persons shall pay according to their means." This practically opens the hospitals of the zone as private pay hospitals to all the world, for it would be difficult for any sick persons to come from anywhere and to enter the zone at either end without being sick in Colon and Panama. As a matter of fact, it happens with increased frequency that patients, not only from Panama, but from various points along the coast and in the interior, are received and treated in the hospitals at Ancon. I have no knowledge touching this point at Colon, but can see no reason why it should not be equally true there.

The thing, however, would not seem so bad if the provision that these patients should pay according to their means had been permitted to stand without qualification. Unfortunately, however, the commission, more especially Mr. Grunsky, with his fondness for prescribing details, proceeded to lay down a per diem charge for room and for nursing at Ancon, the rates being about the same as those charged for similar service in the better hospitals of the United States. He, however, did not stop at this point, but made it a part of the laws of the canal zone that "surgical operations shall be charged for according to their importance and the financial ability of the patient to pay, but no charge in excess of $50 shall be made without the approval of the director of hospitals." As the office of director of hospitals has been abolished, the maximum rate to be charged for surgical operation, without reference to their gravity, stands at $50. It is furthermore provided by Mr. Grunsky and made part of the laws of the canal zone, that physicians connected with the hospitals must visit the residences of persons "entitled to treatment at the hospital, at a fixed daily rate of pay," which visits "shall be charged for at the rate of one-half dollar ($0.50) for each visit, but no such charge is to be made for emergency calls nor in the cases of persons entitled to free hospital treatment until after the person has been ordered into the hospital." These rates which, in the instance of surgical operations, vary from one-fourth to one-tenth of the average charge in the United States and in the Republic of Panama, and in the instance of visits, less than one-fourth of the ordinary rates charged in either of the two countries, cannot but be disgusting to the medical profession in the states who are not in the least influenced by the belittling competition.

It is important, however, to bear in mind that the physicians at Ancon are in no sense the beneficiaries of the extra revenue that is derived from the extra service thus imposed on them. On the contrary, it is provided by the commission, more especially by Mr. Grunsky, and duly engrossed in the laws of the canal zone that:

> The money realised in the hospitals from charges for board, medical attendance, surgical operations and the like, and from visits at residences as set forth, shall be considered public funds and turned over to the government of the canal zone.

It follows, therefore, that it is not the medical men sent to the canal zone, but the United States, our own great nation itself, that is engaged in this belittling business, which must be recognised as a personal insult to any decent practitioner of medicine in any country of the world.

In the Republic of Panama it amounts to more than an insult; it is an actual damage. This was brought to my attention by one of the most distinguished members of the medical profession of that country, who first made me aware of the situation, and who did so in terms of bitter complaint. He explained that under the operation of this order of things, and in spite of the existence of other hospitals in Panama, private patients, attracted by the cheap rates and the prestige of the medical staff at Ancon, are flocking to that institution to the present serious embarrassment and prospective ruin of medical practitioners.

I took the liberty to mention this matter in the course of a personal interview with President Amador, himself the most distinguished medical practitioner of the republic. As he is a friend of long standing, I had no hesitancy in broaching the subject and no difficulty in securing from him a very frank expression of fact that the situation had become so serious that he had been waited on by his colleagues of the medical profession, his constituents and fellow-citizens, by whom he had been requested to make formal representations on the subject to the American minister, but that he had desisted out of his great desire to avoid even the semblance of friction with the United States, and in the belief that a knowledge of so flagrant an injustice would sooner or later reach executive circles in Washington and would be corrected.

But a commission that is capable of thus busying itself with petty but, in their hands, annoying and obstructive details, would naturally enough be expected to forget matters of real importance. One cannot, however, even at the hazard of seeming impertinent, enquire: "Why is there yellow fever in Panama?"

### WHY IS THERE YELLOW FEVER IN PANAMA?

Yellow fever is demonstrably a preventable disease, and as a consequence all deaths resulting from that disease must at once raise the question of responsibility. Panama has long and justly been recognised as a seat of yellow-fever infection, just as was Havana before the brilliant sanitary achievements of Colonel Gorgas in that city,—achievements which resulted in his call to the Isthmus. The real campaign against the disease in Havana, more particularly against the disease-bearing mosquitoes, lasted from January to September, 1901. As Panama is but a village in comparison with the Cuban metropolis, it was naturally expected that similarly satisfactory results would be realised there in the same if not less time. But yellow fever is still endemic in Panama. Why is this true?

The dangers of the situation were thoroughly appreciated by Colonel Gorgas, even before he went to the Isthmus, and he laid before the commission a plan of campaign which embraced several distinct features—namely:

1. The installation of a sewer system in the cities of Colon and Panama.
2. The installation of water supply in those cities.
3. The cleaning of the streets including the disposal of garbage and night soil.
4. General sanitation of houses including their fumigation and the drainage of neighboring pools, the abolition of water barrels and cisterns and other places for the propagation of the yellow fever mosquito.
5. The prompt isolation of all cases of yellow fever.

These various steps bore such a logical relation to each other that one would become practically inoperative without the other. Thus, without drainage, a water supply would only augment the evil; without both it would be impossible to abolish the cisterns and water barrels that are breeding places of the yellow-fever mosquitoes; without abolishing the breeding places of these mosquitoes, it would be useless to attempt the formation of more than demonstrably infected houses.

This view was apparently accepted by the commission, even including Mr. Grunsky, before the first visit of that body to the Isthmus, and it was likewise the view still held by Colonel Gorgas and by the entire personnel of the sanitary department, as well as by Mr. Wallace, the head of the engineering department, when, on June 28, the sanitary government was established in the canal zone. Panama was then apparently free from yellow fever, but Colonel Gorgas, with his Cuban experience, and knowing the danger that was lurking in the immediate future, set about promoting these complete measures of prevention, while Mr. Wallace addressed himself to plans and specifications for water-works and sewer system for Panama. The plans of both of these men went promptly before the commission, but it was not until that body had returned to the Isthmus, in August following, that they were given serious consideration; meanwhile, the danger of which Colonel Gorgas had forewarned them had developed; for, on July 12, Charles Cunningham was stricken with yellow fever, from which he died two days later. Fourteen days after this death another case developed, which, however, went on to recovery. When the commission arrived at Ancon on the occasion of its second visit, that is, on August 3, Colonel Gorgas again urged the prompt assumption of sanitary control over Colon and Panama, and cited the case still in the hospital, and the fatal one that had preceded it, as danger signals of sufficient gravity to justify action. But the commission, more especially Mr. Grunsky, had not yet determined the degree of humiliating subordination to which the sanitary department was to be subjected, and, under pretext of perfecting a plan of organisation, deferred action for another three and one-half weeks,—valuable weeks,—that is, until August 28. On this date the commission, on the report of Mr. Grunsky, adopted Mr. Grunsky's plan of organisation, by which, as I have already shown, Colonel Gorgas was subordinated to the seventh degree below the original source of authority. Even then, with the cases of yellow fever staring them in the face, the commission, at the instance of Mr. Grunsky, directed Colonel Gorgas, acting through Governor Davis, to refrain from any attempt to secure sanitary control over the cities of Colon and Panama, citing certain more or less diplomatic frivolities as a pretext for deferred action. It was only after four or five months had elapsed, only after the progressive development of yellow fever had reached the sensational point, and only after the personnel in the canal zone had become thoroughly alarmed over the situation, that Colonel Gorgas was permitted by those in authority over him to assume the sanitary control of the two cities, one of which, Panama, having by this time become very generally infected.

But even then his hands were tied, sometimes, and in important particulars, by the arbitrary exercise of superimposed authority, but all the time, and in still more important particulars, by the fact that the water supply and the sewage system were not installed. Mr. Wallace had drawn the plans and specifications in July previous, and had taken care to specify only such pipe as manufacturers keep in stock, and that could therefore be procured without a moment's delay. But the commission, more especially Mr. Grunsky, in total disregard of the emergency that was present, saw fit to indulge in some views about pipe, and as Mr. Grunsky is a civil engineer and needed to impress the fact on somebody, he summoned Mr. Wallace, confessedly one of the ablest engineers but now his subordinate, before the commission to explain why he had specified both eastern and western standards of pipes. The explanation given by Mr. Wallace that either one or both of these standards would answer the purpose, and that it had been simply his desire to purchase the pipe promptly, if necessary in small lots, and to get the pipe promptly on the Isthmus, seemed to make no appeal to either the commission or Mr. Grunsky, and pipe of the western pattern alone was ordered. The delay in the consideration, of this particular point consumed another precious two weeks, after which Mr. Wallace was authorised to proceed with the work. This he undertook with his characteristic energy, and allowing for all reasonable delay in procuring the pipe and in sending it to the Isthmus, promised the people of Panama that they should have water by December. He finished the work at the Rio Grande reservoir in a short time; the trenches for the pipe were dug, washed full of dirt and redug, but still there was no pipe. Mr. Wallace then cabled to Washington, urging that pipe be sent, only, however, to receive a reprimand from Admiral Walker, chairman of the commission, admonishing him that cablegrams from the Isthmus were expensive. It is now nearly the first of March, and the schooner which brought the first consignment of pipe, not enough to complete the work at Panama, was discharging the cargo at Colon as I left. It is further understood that before the work can go on this same schooner must sail back to Mobile, await the arrival there of enough pipe to make a full cargo, then sail back to Colon and again unload—a proceeding that, at a conservative estimate, will consume at least from two to three months. And all this by a commission that controls a line of steamers plying weekly between New York and the Isthmus!

In the light of all these facts, in contrast with the brilliant results achieved by Colonel Gorgas in Havana, where he was given not only a free hand, but his own purchasing and distributing agents, the responsibility for the present existence of yellow fever on the Isthmus can be placed nowhere else than on the Isthmian Canal Commission, more especially on Mr. Grunsky.

### THE CAMPAIGN AGAINST MALARIA THWARTED BY THE COMMISSION.

But sensational as is yellow fever, now apparently on the increase at the Isthmus, the fact remains that malaria is the more serious pest. The discovery that malaria is disseminated by the anopheles has, however, made it possible to minimise the disease by proper preventive methods. These embrace (*a*) drainage, (*b*) the isolation of malarial patients, (*c*) the prophylactic synchronism. That these three things might be done, and done before the onset of the dry season, Colonel Gorgas asked the commission last August to furnish men to dig ditches and drain pools about the houses of natives; he asked also for wire screening with which to isolate all patients afflicted with malaria, and quinin with which mildly to cinchonise laborers and others exposed to the malaria bearing mosquitoes. The commission, more especially Mr. Grunsky, however, thought that the number of ditches asked for, something like twenty, was too many, and allowed only half those estimated, but as the dry season has arrived and is almost past, and as the small squad of ditchers is still digging, it would seem that the original estimate was justified by the necessities of the situation. The wire screening was likewise cut down from 2,500 yards to 500 yards, and even that has not yet arrived on the Isthmus. The use of quinin as a preventive of infection did not commend itself either to the great therapeutic skill or to the economic judgment of the commission, more especially Mr. Grunsky, so the observation of medical men with previous experience on the Isthmus, the conclusions of authorities in tropical medicine, such as Munson and Giles, the investigations of the Italian authorities, and the unequivocal verdict of Koch, were pushed aside and quinin for this purpose was stricken from the list.

The result is that while much has been accomplished by drainage, and by the admission of small fish into the runnels made by the small force of workmen, and while much has been accomplished by the prophylactic use of quinin by the personnel and employes of the higher grades, and taken by them in spite of the action of the commission, the fact remains that in the absence of the wire screening ordered last August, each case of malaria becomes a focus for the further dissemination of the disease. This is shown even at the Ancon Hospital, where in the complete absence of wire screening for the malarial wards and the consequent impossibility of excluding the malarial-bearing mosquitoes, non-malarious patients are becoming malarious, while both nurses and attendants are being made frequent victims of the disease. Two members of the medical staff likewise were suffering from malarial infection during the time of my stay at Ancon. Yet in spite of these facts the Isthmian Canal Commission, in its report under date of December 1, 1904, page 88, states that "all cases of employes sick with malaria are taken into the Colon and Ancon hospitals and so screened that mosquitoes cannot reach them"—a statement that is an absolute and an unqualified falsehood.

### CHEAP NURSING SERVICE.

This report might be indefinitely amplified, but time will not permit. I feel it important, however, to allude to the fact that the policy which the commission, more especially Mr. Grunsky, has adopted with reference to furnishing cheap medical service to those who risk their lives in the zone has been adopted for the purpose of furnishing nurses for service in the sanitary department. The effort has been made under the subterfuge of establishing a training school to be conducted at Ancon, to get nurses to go to the zone at about the same rate that is paid for pupil nurses in the training schools of the United States. The same conditions, practically, are imposed on the nurses with reference to time service that is imposed on the internes, with the difference, however, that the period of enforced detention on the Isthmus under contract is placed at three years instead of one. This is not a place to take untrained nurses under any pretext, for nothing but fully developed talent in the various departments of activity should be sent to the Isthmus.

### CONCLUSION.

In view of the foregoing facts and disregarding equally serious facts relating to the department of engineering and construction, facts which the really valuable services of such individual commissioners as Messrs. Parsons and Burr could not entirely neutralise, but on which I do not feel qualified or authorised to report, I beg leave to call attention to the further observations of the President when he inducted the commission into office, as follows:

> I believe that each one of you will serve not merely with entire fidelity, but with the utmost efficiency. If at any time I feel that any one of you is not rendering the best service which it is possible to procure, I shall feel called on to disregard alike my feelings, and forthwith to substitute for him on the commission some other man whom I deem capable of rendering better service.

In view of the facts which I have presented, in view of many more facts of similar purport which might be presented, in view of the President's manifest and expressed wishes and their complete disregard by the commission, but more particularly in view of the vital interests at stake, I have the honor not only to submit the suggestion, but really to urge that the time has arrived when the President ought to redeem his word and ask for the resignation of the commission.

I have the honor to be, very respectfully,

CHARLES A. L. REED,
*Chairman of the Legislative Committee of the American Medical Association.*

